# Investigating the Readability and Linguistic, Psychological, and Emotional Characteristics of Digital Dementia Information Written in the English Language: Multitrait-Multimethod Text Analysis

**DOI:** 10.2196/48143

**Published:** 2023-10-25

**Authors:** Margi Engineer, Sushant Kot, Emma Dixon

**Affiliations:** 1 Computer Science Department Clemson University Clemson, SC United States; 2 Human Centered Computing Department, Clemson University Clemson, SC United States

**Keywords:** natural language processing, consumer health information, readability, Alzheimer disease and related dementias, caregivers

## Abstract

**Background:**

Past research in the Western context found that people with dementia search for digital dementia information in peer-reviewed medical research articles, dementia advocacy and medical organizations, and blogs written by other people with dementia. This past work also demonstrated that people with dementia do not perceive English digital dementia information as emotionally or cognitively accessible.

**Objective:**

In this study, we sought to investigate the readability; linguistic, psychological, and emotional characteristics; and target audiences of digital dementia information. We conducted a textual analysis of 3 different types of text-based digital dementia information written in English: 300 medical articles, 35 websites, and 50 blogs.

**Methods:**

We assessed the text’s readability using the Flesch Reading Ease and Flesch-Kincaid Grade Level measurements, as well as tone, analytical thinking, clout, authenticity, and word frequencies using a natural language processing tool, Linguistic Inquiry and Word Count Generator. We also conducted a thematic analysis to categorize the target audiences for each information source and used these categorizations for further statistical analysis.

**Results:**

The median Flesch-Kincaid Grade Level readability score and Flesch Reading Ease score for all types of information (N=1139) were 12.1 and 38.6, respectively, revealing that the readability scores of all 3 information types were higher than the minimum requirement. We found that medical articles had significantly (*P*=.05) higher word count and analytical thinking scores as well as significantly lower clout, authenticity, and emotional tone scores than websites and blogs. Further, blogs had significantly (*P*=.48) higher word count and authenticity scores but lower analytical scores than websites. Using thematic analysis, we found that most of the blogs (156/227, 68.7%) and web pages (399/612, 65.2%) were targeted at people with dementia. Website information targeted at a general audience had significantly lower readability scores. In addition, website information targeted at people with dementia had higher word count and lower emotional tone ratings. The information on websites targeted at caregivers had significantly higher clout and lower authenticity scores.

**Conclusions:**

Our findings indicate that there is an abundance of digital dementia information written in English that is targeted at people with dementia, but this information is not readable by a general audience. This is problematic considering that people with <12 years of education are at a higher risk of developing dementia. Further, our findings demonstrate that digital dementia information written in English has a negative tone, which may be a contributing factor to the mental health crisis many people with dementia face after receiving a diagnosis. Therefore, we call for content creators to lower readability scores to make the information more accessible to a general audience and to focus their efforts on providing information in a way that does not perpetuate overly negative narratives of dementia.

## Introduction

### Background

Seeking and obtaining web-based health information [1] is a critical component of health and disease management [2-7]. This becomes particularly important for conditions where information is not readily provided by clinicians upon initial diagnosis, as is common when people are diagnosed with dementia in Western societies [8-10]. Further, the limited information provided at diagnosis is often conveyed in an overly negative manner [11-14].

Owing to the lack of information provided at diagnosis [15], as well as the overwhelmingly negative nature of the limited information provided, in Western societies, after receiving a dementia diagnosis, people living with dementia [16-18] and their care partners [11,19-22] use various information-seeking methods to search for web-based dementia information to re-establish a sense of well-being and emotional balance [8]. This often includes reading web-based medical articles with the latest breakthroughs in clinical research and reviewing and subscribing to large dementia advocacy and medical organization websites and electronic newsletters [8,23,24]. In addition, people living with dementia and informal caregivers use other web-based information mediums where people write and share their personal experiences living with or caring for people living with dementia, such as Twitter [6,25,26], web-based forums [27-31], and dementia-specific websites and blogs [32,33]. These alternatives to medical articles and information from advocacy organizations provide space for shared experiences, exchanges of information, and emotional support [28,34-38].

Although many people living with dementia [13,32] and their caregivers [20,21,39,40] search for digital dementia information in the ways previously described, there remain barriers and challenges to these methods. Accessibility of digital dementia information is one such barrier reported by people with dementia and informal caregivers. This includes the emotional inaccessibility of medical articles and dementia websites owing to overly pessimistic wording and a focus on end-of-life preparation [32] as well as the cognitive inaccessibility [41,42] of digital dementia information owing to excessive word count and complicated language [32,43]. Another barrier reported by individuals with dementia is a lack of information relevant to them, as opposed to the information written for a target audience of caregivers or clinicians [32]. Yet another barrier is the reliability and accuracy of digital dementia information shared in web-based forums, peer support groups, and other informal forms of peer-to-peer web-based information sharing [28,29,44].

In response to the concern regarding the accuracy of digital dementia information, researchers have begun to investigate the eHealth literacy of people with dementia and their informal caregivers [28,44-50]. eHealth literacy refers to the ability to appraise health information from electronic sources and apply the knowledge gained to addressing or solving a health problem [51]. This past research found that lower education levels [44] and older age [47] of caregivers were associated with lower health literacy and lower levels of dementia knowledge [44]. Lower health literacy of caregivers is associated with lower caregiving self-efficacy [45] and, consequently, lower health outcomes for the person with dementia they care for [47]. In addition, researchers have shown that lower health literacy in older adults is correlated with a higher risk of dementia [48]. Therefore, it is imperative to develop digital dementia informational resources that are responsive to the low eHealth literacy levels of this population [46].

Taken together, this past research describes ways in which people with dementia and their caregivers search for digital dementia information, the challenges associated with this population searching for digital dementia information, and the need for further work to make digital resources more accessible to this population. In this study, we lay the groundwork for future research investigating ways to design dementia resources that better meet the eHealth literacy and accessibility needs of people with dementia and their informal caregivers by examining the readability; linguistic, psychological, and emotional characteristics; and target audiences of 3 types of digital dementia information written in the English language.

### Objectives

This study had 2 primary aims. The first aim was to investigate the availability of web-based information for different target audiences. We conducted a thematic analysis of 612 web pages from 35 dementia advocacy and medical organization websites as well as 227 unique blog posts from 50 dementia-specific blog sites to investigate the percentage of unique informational sources targeted at an audience of people with dementia or caregivers or a general audience. The 300 medical articles collected were assumed to be targeted at an audience of clinical practitioners and were, therefore, categorized into those that were written with more biological and neuroscience terminology and those written with less medical terminology.

The second aim of this study was to investigate the emotional and cognitive [52] accessibility of three types of web-based dementia information formats, namely (1) blogs written about dementia, (2) dementia advocacy and medical organization websites, and (3) dementia medical articles, using natural language processing (NLP) tools. Researchers [53-56] are increasingly developing and using NLP tools [24,53,57] in health care settings, such as to predict and analyze cognitive functions [16,58-62] through advanced processing of textual and speech data of patients, which may include medical records, patient history, physician’s notes, and even conversations between health care providers [17,60] and patients. In this study, we incorporated NLP techniques to perform sentiment analysis and gauge the tone of the text, as was done in a study that analyzed the readability of digital Parkinson disease information [63] and to analyze different textual formats of general health information [1]. To measure readability [64], we performed Flesch Reading Ease and Flesch-Kincaid Grade Level [65] analyses. To measure features that reflect different psychological [40,58,66,67] and emotional processes embedded in the text, we performed a Linguistic Inquiry and Word Count (LIWC) [68-70] analysis. We sought to compare the readability and LIWC scores of diverse types of digital dementia information and the categories [67] within these different types of information to investigate the accessibility of digital dementia information.

## Methods

### Data Collection and Exclusion

In the data collection process, the first and second authors used a search engine to find 612 web pages from 35 dementia advocacy and medical organization websites as well as 227 unique blog posts from 50 dementia-specific blog sites, which were used in this analysis. They then went through each web page of the websites and blogs to scrape information related to dementia and store it in Excel (Microsoft Corp). From each blog and website, they scraped only the body text and informational content. Therefore, titles, subtitles, references, web links, advertising text, website log-in pages for web-based forums, and contact information of specific organizations or retirement homes were not considered. Data from websites or blogs that contained only contact details as information were not included.

Advocacy websites with information related to dementia or with headings that indicated that the information was for those living with dementia or for caregivers were selected. This included web pages from organizations such as Dementia Australia [71], the Alzheimer’s Society Canada [72], the United Kingdom Alzheimer’s Society [73], and the United States Alzheimer’s Association [50]. We excluded e-commerce websites related to dementia and advertising websites from the list. Although some of these websites may offer downloadable content, the primary focus of this study was to analyze digital information. As a result, leaflets and downloadable data were excluded from our selection.

Blogs that offered information about dementia or care partners of people living with dementia, such as Early Onset Alzheimer’s [74], Life With Father-Navigating Parental Caregiving in First Person [75], and As Our Parent Age So Do We [76], as well as informational content written and shared by individuals living with dementia (eg, Dementia Diaries [77], Living with Dementia and Comorbidities [78,79], and Which Me Am I Today [80]) were selected. In addition, some blogs authored by dementia health care professionals were also included in the selection (eg, Finding the Light in Dementia [81], Dementia by Day [82], and James L West Center for Dementia Care [83]).

PubMed was used to collect 300 medical articles. Only medical articles that were open access and published within the last 5 years were included, as past work found that people with dementia were filtering their searches to find more recently published articles and those that were freely available to download [8]. In addition, only papers that reported using the following methods were included: randomized control, meta-analysis, clinical trial, review, and systematic review. The authors intentionally covered a wide range of article topics to reflect the multifaceted nature of dementia and the diverse interests and concerns of scientists who write about it. Although this diversity makes it challenging to categorize the articles by topic area, it provides a more holistic sample of the readability [84] and linguistic characteristics of the dementia literature people may come across. The medical articles excluded from consideration were those that were highly technical with language that was filled with medical jargons (eg, the studies by Arai et al [85], Chang et al [86], Gauthier et al [87], and Ma et al [88]). Further, only articles published in verified publications such as Elsevier, PubMed, Nature, and IEEE were selected for inclusion. The PDFs of the 300 most recently published medical articles that fit the filtering criteria were downloaded and saved in a shared folder among the authors. A list of the medical article titles in alphabetical order was stored in an Excel sheet to organize the analysis.

### Thematic Analysis

To categorize the data, a thematic analysis method [89] was followed. Each web page and blog was read and organized according to the target audience of the information source: *people with dementia* [12,17,26,29,90-93], *caregivers* [20-22], or a *general audience* [33,94,95]. Often, the target audience of an informational source was made clear in the web page title or introductory paragraphs of the content. For example, web pages titled “How to Parent Your Parent Who Has Alzheimer,” “Living with Alzheimer Disease,” and “CaringKind Alzheimer’s Walk” would be categorized as targeting caregivers, people with dementia, and a general audience, respectively. If the target audience was not clear in the title or introductory paragraph of the informational resource, then the full resource was read and categorized. These categories were used for the following two primary reasons: (1) to investigate claims from past work of a lack of information relevant to people living with dementia [32] and (2) to avoid organizing and analyzing informational resources by the organization or persons who published them, as the goal of this study was not to identify specific organizations that published accessible or inaccessible information resources.

Owing to the vast range of topics covered in the articles and the fact that the aim was not to conduct a literature review, the articles were not categorized based on the topic of study. Instead, medical articles were characterized as those that included more biological and neuroscience terminology (eg, “Dengzhan Shengmai Capsule Combined With Donepezil Hydrochloride in the Treatment of Alzheimer’s Disease: Preliminary Findings, Randomized and Controlled Clinical Trial” [96]) and those that had less medical terminology (eg, “Characteristics and Value of ‘Meaningful Activity’ for People Living With Dementia in Residential Aged Care Facilities: ‘You’re Still Part of the World, Not Just Existing’” [1]).

### Readability Assessment

The readability [97] of digital content from each of the websites, blogs, and medical articles was analyzed using 2 validated measures: the Flesch-Kincaid Grade Level and Flesch Reading Ease [98,99], each of which available through Word (version 16.7; Microsoft Corp). Full content from web pages and body text from blogs were extracted into Word documents and analyzed. All medical articles were saved as PDFs; opened as Word documents; and analyzed in their entirety, including titles, subtitles, authors, body text, and references.

The Flesch-Kincaid Grade Level measure analyzes the digital content, producing a score that estimates the US school grade level required to understand the text using the following formula: ([0.39 × average sentence length] + [11.8 × average syllables/word] −15.59) [65,98,99]. Most text-based content aims for a score of approximately 7.0 to 8.0 [65,100], meaning that one would need to complete their seventh to eighth grade of education to understand the content.

The Flesch Reading Ease measure analyzes the digital content, producing a score that estimates the ease at which the text will be understood using the formula: 206.835 − (1.015 × average sentence length) − (84.6 × average number of syllables/word). This test rates text on a scale of 0 to 100 points [101]. The higher the score, the easier it is to understand the text; most content aims for a score between 50 and 70, meaning that the text is between fairly difficult to read (50-60) and plain English (60-70) [65,100,101]. Text scoring between 50 and 30 is considered difficult to read, and some college education would likely be required to understand it [101]. Text scoring between 30 and 10 is considered very difficult to read and would best be understood by college graduates [101]. Text scoring between 10 and 0 is considered extremely difficult to read and would best be understood by college graduates [101].

### LIWC Analysis

#### Overview

The LIWC [62,98,102] system uses a dictionary that categorizes psychological characteristics [66,67] into specific words that convey those attributes [103]. The LIWC-22 [68] comprises >100 preexisting dictionaries that are designed to identify various psychological [66,67] and social states of individuals. These dictionaries consist of a range of lexical items, including words, emoticons, and other specific verbal expressions that are indicative of a particular psychological category. LIWC analyzes a given text by comparing each word in it with the dictionary words and then calculating the proportion of words that belong to each category. The scores are standardized and converted into percentiles ranging from 1 to 99 (based on the area under the normal curve). Generally, the score for a particular category can range from 0 to 100, where 0 indicates that no words in the text were coded for that category, and 100 indicates that all words in the text were coded for that category. The LIWC analytic scores may differ based on the text’s length and the category under analysis. However, the low score range is generally between 0 and 20, whereas the high score range is usually between 80 and 100, [68,69,103].

In this study, the LIWC-22 dictionary was used to analyze the 5 main parameters of LIWC: word count, analytical thinking, clout, authenticity, and emotional tone, which are described in the subsequent subsections. In addition, meaning extraction analysis from LIWC, which shows the word frequencies (ie, how many times each word was used in the data), was used to further investigate the emotional status of the collected data.

#### Word Count

The LIWC software uses linguistic and psychological dimensions to categorize words and word stems for word count analysis [104,105]. The overall word count for each information type and the most frequently used words within each information type were investigated. In addition, the number of unique informational resources that included specific categories of the LIWC dictionary were investigated: positive emotion (words such as “happy” and “love”), negative emotion (words such as “sad” and “hate”), positive tone (words such as “benefits,” “care,” “improving,” and “caring”) and negative tone (words such as “death” [106], “depression,” “cognition,” “impairments,” and “psychosocial”). Within each of these larger categories, there are subcategories that constitute the larger scores. Of specific interest was the number of unique informational resources that used words related to “wellness,” “anxiety,” “anger,” “sad,” “illness” [107], and “death.” The frequency analysis conducted through LIWC is a valuable tool for understanding the words used in a piece of text and their overall prominence in the content.

#### Analytical Thinking

Analytical thinking [108-111] gauges the extent to which individuals use language that suggests formal, logical, and hierarchical modes of thinking. People who score low in analytical thinking (range) tend to use language that is more personal and intuitive, whereas individuals who score high tend to use language that is more structured and logical.

#### Clout

Clout [6,112,113] is a measure of the level of social status, confidence, or leadership that individuals exhibit in their writing or speech. The clout algorithm was developed based on the findings of a series of studies in which people engaged in social interactions (eg, the study by Kacewicz et al [112]). It is important to note that clout differs from the concept of “power” (which is represented by the LIWC-22 “power” variable). Power, or the desire for power, reflects an individual’s attention to or awareness of their social status within a particular context.

#### Authenticity

The authenticity [114] algorithm was initially established through a series of studies in which participants were prompted to be truthful or deceitful [115] as well as a summary of studies on deception published in subsequent years [70]. However, as time has passed, it has become apparent that the authenticity measure is less concerned with “deception” in the conventional sense and is more indicative of an individual’s degree of self-monitoring. Texts that score low in authenticity include prepared speeches and cautious social interactions, whereas texts that score high in authenticity tend to be spontaneous conversations between close friends or influential individuals with few social inhibitions.

#### Emotional Tone

Although LIWC-22 comprises both positive and negative tone dimensions [110,116,117], the tone variable consolidates the 2 dimensions into a single summary metric. A high median score in the emotional tone measure could suggest that the text has a highly emotional or expressive tone [118], which could be indicative of creative writing, personal narratives, or persuasive arguments that appeal to the emotions of the reader. However, it is important to note that a high median score in emotional tone does not necessarily indicate whether the emotions expressed in the text are positive or negative or whether they are appropriate in a given context.

### Statistical Analysis

The 5 main LIWC categories as well as readability scores were analyzed in SPSS (IBM Corp) as dependent variables. For each variable, the Kolmogorov-Smirnov test [119] was used to test for normality in each distribution. When data were found to follow a normal distribution, means and 95% CIs were provided; otherwise, we provided medians with 95% CIs. Further, Kruskal-Wallis tests [120,121] were used to compare the LIWC categories as well as readability scores among the different types of information sources (advocacy websites, blogs, and medical articles) as well as the different audiences of the combined websites and blogs (caregivers, people with dementia, and a general audience). A Mann-Whitney test was performed to compare the LIWC categories and readability scores between the different types of medical articles (biological specific and other). Statistical significance [122] was set to .05.

### Ethical Considerations

All digital dementia information used in this study was collected from publicly available sources on the web, meaning that anyone with internet access can view this content at any time. An individual does not need to be a member of an organization to log into an account to access any of the digital information used in this analysis. Therefore, according to US federal regulations, this project does not meet the definition of human participant research and is not under the purview of the institutional review board.

## Results

### Information Target Audience

In total, 1139 unique dementia informational sources were analyzed, including 227 (19.93%) blog posts, 612 (53.73%) web pages of dementia advocacy websites, and 300 (26.34%) research articles. Among the 227 blog posts analyzed, 45 (19.8%) were categorized as targeted at caregivers, 26 (11.5%) were categorized as targeted at a general audience, and 156 (68.7%) were categorized as targeted at people living with dementia. Of the 612 web pages collected and analyzed, 74 (12.1%) were targeted at caregivers, 139 (22.7%) were targeted at a general audience, and 399 (65.2%) were targeted at people with dementia. Among the 300 medical articles, 72 (24%) were categorized as reporting research related to biology and neuroscience, and 228 (76%) were categorized as reporting clinical research more generally.

### Readability

#### Overview

**Figure 1:**
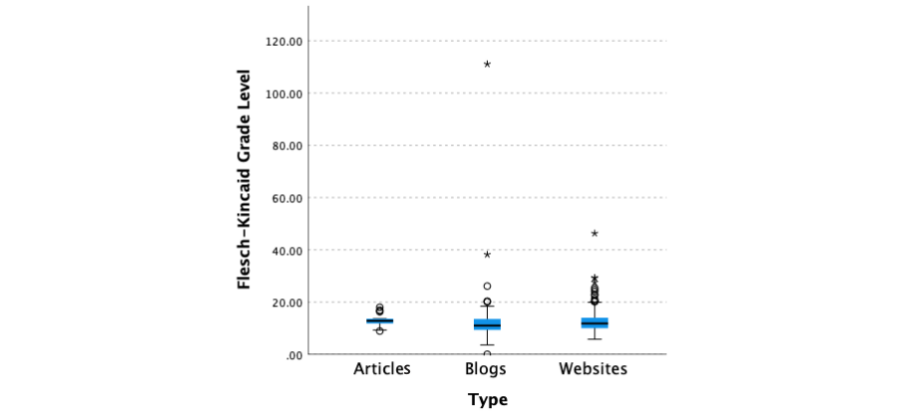
Flesch-Kincaid Grade Level scores by information type.

**Figure 2:**
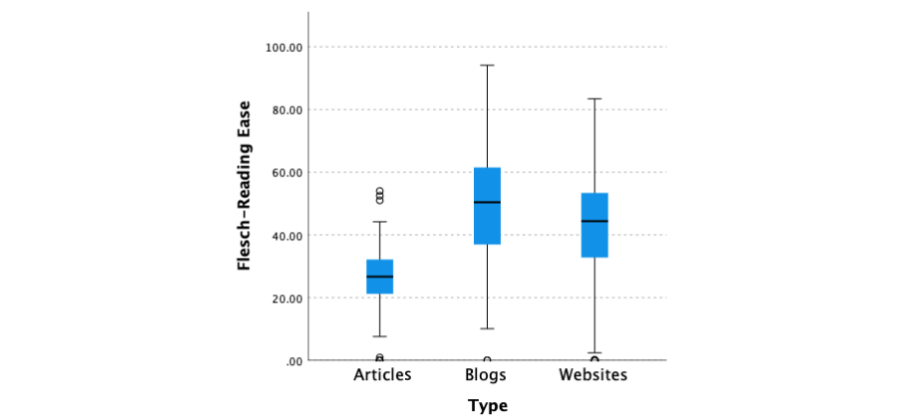
Flesch Reading Ease scores by information type.

The median readability score across all information types (N=1139) for Flesch-Kincaid Grade Level was 12.1 (95% CI 11.9-12.3; Figure 1), and the median Flesch Reading Ease score was 38.6 (95% CI 36.7-40.7; Figure 2). A Kruskal-Wallis test was performed to compare Flesch Reading Ease and Flesch-Kincaid Grade Level scores across the information types, namely websites, blogs, and medical articles. There was very strong evidence of a difference (*P*<.001) between the distributions of at least one pair of target audiences for both Flesch Reading Ease and Flesch-Kincaid Grade Level scores. Wilcoxon signed rank pairwise tests were performed for the 3 information types. There was extraordinarily compelling evidence (*P*<.001, adjusted using the Bonferroni correction) of differences in the Flesch Reading Ease scores among websites, blogs, and medical articles. The median Flesch Reading Ease score for medical articles (300/1139, 26.34%) was 26.7 (95% CI 25.2-27.8), which was substantially lower than those for blogs (50.4, 95% CI 48.2-52.3; 227/1139, 19.93%) and websites (44.3, 95% CI 43.1-45.6; 612/1139, 53.73%). There was very strong evidence (*P*<.001, adjusted using the Bonferroni correction) of differences in the Flesch-Kincaid Grade Level scores among medical articles (12.8, 95% CI 12.6-13.0; 300/1139, 26.34%), blogs (11.00, 95% CI 10.5-11.5; 227/1139, 19.93%), and websites (11.8, 95% CI 11.5-12.1; 612/1139, 53.73%). There was also evidence (*P*=.01, adjusted using the Bonferroni correction) of a significant difference between blogs (11.0, 95% CI 10.5-11.5; 227/1139, 19.93%) and websites (11.8, 95% CI 11.5-12.1; 612/1139, 53.73%).

#### Websites

The median readability score across all websites (n=612) for Flesch-Kincaid Grade Level was 11.8 (95% CI 11.5-12.1), and the median Flesch Reading Ease score was 44.4 (95% CI 43.1-45.6). A Kruskal-Wallis test was performed to compare Flesch Reading Ease and Flesch-Kincaid Grade Level scores across website target audiences, namely people with dementia, caregivers, and a general audience. There was very strong evidence of a difference (*P*<.001) between the distributions of at least one pair of target audiences for both the Flesch Reading Ease and Flesch-Kincaid Grade Level scores. Wilcoxon signed rank pairwise tests were performed for the 3 groups of target audiences. There was extraordinarily convincing evidence (*P*<.001, adjusted using the Bonferroni correction) of differences in the Flesch-Kincaid Grade Level and Flesch Reading Ease scores among a general audience, caregivers, and people with dementia. The median Flesch-Kincaid Grade Level and Flesch Reading Ease scores for the information targeted at a general audience (144/612, 23.5%) were 12.6 (95% CI 12-13.4) and 38.5 (95% CI 35.9-40.8), respectively. These scores were significantly lesser than those of the information targeted at caregivers (70/612, 11.4%)—Flesch-Kincaid Grade Level score of 10.55 (95% CI 10-11.5) and Flesch Reading Ease score of 48.4 (95% CI 45.9-53.6)—and information targeted at people with dementia (398/612, 65%)—Flesch-Kincaid Grade Level score of 11.55 (95% CI 11.2-12) and Flesch Reading Ease score of 45.2 (95% CI 43.8-46.7). There was no significant difference in Flesch Reading Ease (*P*=.19, adjusted using the Bonferroni correction) and Flesch-Kincaid Grade Level (*P*=.15, adjusted using the Bonferroni correction) scores between information on websites targeted at caregivers and information on websites targeted at people with dementia.

#### Blogs

The mean Flesch Reading Ease score for all blogs (n=227) was 49.10 (95% CI 46.813-51.386), and the median Flesch-Kincaid Grade Level score was 11.514 (95% CI 10.99-12.03). A Kruskal-Wallis test was performed to compare Flesch Reading Ease and Flesch-Kincaid Grade Level scores for website target audiences, namely people with dementia, caregivers, and a general audience. There was no considerable evidence of a difference between the distributions of any pairs of target audiences for either the Flesch Reading Ease (*P*=.21) or Flesch-Kincaid Grade Level score (*P*=.40).

#### Medical Articles

The mean Flesch Reading Ease score for all medical articles (n=300) was 26.339 (95% CI 25.373-27.304), and the median Flesch-Kincaid Grade Level was 12.8 (95% CI 12.6-13.0). A Mann-Whitney *U* test was performed to compare Flesch Reading Ease and Flesch-Kincaid Grade Level scores for medical articles with a biological focus with those of general medical articles. There was very strong evidence of a difference between the medical articles that had a more biological focus and those that did not for both the Flesch Reading Ease (*P*<.001) and Flesch-Kincaid Grade Level scores (*P*=.004). The mean Flesch Reading Ease score for the biological medical articles (72/300, 24%) was 23.488 (95% CI 21.804-25.17), which was lesser than the mean Flesch Reading Ease score for the less biology-focused medical articles (228/300, 76%), 27.167 (95% CI 26.033-29.3). The median Flesch-Kincaid Grade Level score for the biological medical articles was 13.233 (95% CI 12.878-13.589), which was higher than the median Flesch-Kincaid Grade Level score for the less biology-focused medical articles, 12.614 (95% CI 12.426-12.802).

### LIWC: Sentiment Analysis

#### Word Count

##### Overview

LIWC analysis of text data from 1139 unique informational sources across 3 different information types, namely websites, blogs, and medical articles, found that the median word count was 758 (95% CI 696-820). When analyzing the information types separately, we found that the median word count for medical articles was significantly higher than those for blogs (*P*<.001) and websites (*P*<.001). The median word count of medical articles was 8430 (95% CI 7976-8769), that of blogs was 649 (95% CI 584-684), and that of websites was 452 (95% CI 406-520). Further, blogs had significantly higher word counts than websites (*P*<.001, adjusted by the Bonferroni correction).

Using LIWC, we were able to conduct a frequency analysis of the words in the data we collected, which provided insights into their meaning. In blogs, the word “dementia” had the highest frequency (n=2079), followed by “alzheimer’s” (n=1070), “caregivers” (n=350), and “cognitive” (n=184). In articles, “dementia” was the most frequent word (n=20,344), followed by “care” (n=9284), “cognitive” (n=7386), and “alzheimer” (n=5342). In websites, the most frequency word was again “dementia” (n=7244), followed by “alzheimer” (n=4740), “memory” (n=950), and “life” (n=717). In addition, we observed the frequencies of select LIWC categories that are associated with positive emotions (“emo_pos,” “tone_pos,” and “wellness”) and negative emotions (“emo_neg,” “tone_neg,” “anger,” “sad,” “illness,” and “death”). Table 1 provides a breakdown of the number of unique informational sources by information source type that included words that indicated positive or negative tone and emotion.

**Table 1:** Number of unique sources with Linguistic Inquiry and Word Count (LIWC) subcategory words and average percentage of categorized words per source for each type of analyzed information^a^.

LIWC category	Web pages (n=612), n (%)	Words per web page (%; n=612), mean	Blogs (n=227), n (%)	Words per blogs (%; n=227), mean	Articles (n=300), n (%)	Words per articles (%; n=300), mean
Positive tone	564 (92.2)	4.4	227 (100)	4.1	300 (100)	1.7
Negative tone	584 (95.4)	1.8	227 (100)	1.7	300 (100)	1.2
Emotionally positive words	339 (55.4)	0.7	193 (85)	1	296 (98.7)	0.1
Emotionally negative words	578 (94.4)	1.1	227 (100)	0.9	300 (100)	0.8
Anxiety	222 (36.3)	0.5	137 (60.4)	0.4	235 (78.3)	0.1
Anger	111 (18.1)	0.9	67 (29.5)	0.3	179 (59.7)	0.04
Sad	170 (27.8)	0.4	109 (48)	0.4	294 (98)	0.4
Illness	601 (98.2)	4.9	226 (99.6)	2.6	300 (100)	1.7
Wellness	253 (41.3)	0.8	106 (46.7)	0.7	293 (97.7)	0.3
Death	112 (18.3)	0.4	60 (26.4)	0.3	210 (70)	0.1

^a^For each unique information source that included a subcategory, the percentage of all the words in that source that fell within a specified category was calculated. The average of all these percentages is reported in the table.

##### Websites

A Kruskal-Wallis test was performed to compare word count among the 3 distinct categories of website target audiences, namely people with dementia, caregivers, and a general audience. There was very strong evidence of a difference (*P*<.001) between the distributions of at least one pair of target audiences for word count. Wilcoxon signed rank pairwise tests were performed for the 3 groups for target audiences. There was very strong evidence (*P*<.001, adjusted using the Bonferroni correction) of differences in the word count between information targeted at people with dementia (median 536, 95% CI 462-613) and information targeted at caregivers (median 343, 95% CI 233-407; *P*=.006) or information targeted at a general audience (median 353, 95% CI 278-444; *P*=.01). There was no significant difference in word count between information targeted at caregivers and information targeted at a general audience (*P*=.99), adjusted using the Bonferroni correction.

##### Blogs

There was no significant difference in blog word count across the 3 target audiences (*P*=.48). The overall median word count for blogs was 649 (95% CI 584-684).

##### Articles

There was no significant difference in medical article word count between the 2 types of articles (*P*=.34). The median word count of all articles was 8430.50 (95% CI 7976-8769).

#### Analytical Thinking

##### Overview

**Figure 3:**
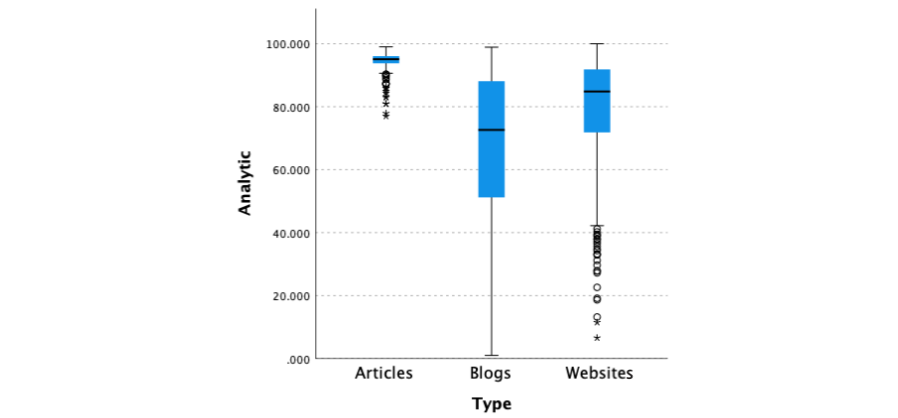
Linguistic Inquiry and Word Count analytical thinking scores by information type.

The median analytical thinking score for all 3 types of data was 88.54, (95% CI 87.33-89.52; Figure 3). There was a significant difference (*P*<.001) in the median analytical thinking scores among the 3 types of data, namely blogs, websites, and medical articles, with medical articles having significantly greater (*P*<.001, adjusted by the Bonferroni correction) analytical thinking scores (median 95.07, 95% CI 94.86-95.27) than blogs (median 72.61, 95% CI 68.94-77.91) and websites (median 84.840, 95% CI 83.610-86.210). Further, websites had significantly higher analytical thinking scores than blogs (*P*<.001, adjusted by the Bonferroni correction).

##### Websites

The distribution of the analytical thinking scores of websites was not significantly different across the 3 target audiences, as confirmed by an independent sample Kruskal-Wallis test (*P*=.23). The median score for all web pages (n=612) was 84.840 (95% CI 83.610-86.210).

##### Blogs

The distribution of the analytical thinking scores of blogs was not significantly different across the 3 target audiences, as confirmed by an independent sample Kruskal-Wallis test (*P*=.94). The median score for all blogs (n=227) was 72.61 (95% CI 68.94-77.91).

##### Articles

The distribution of analytical thinking scores of medical articles was not significantly different across the 2 types of medical articles, as confirmed by an independent sample Mann-Whitney *U* test (*P*=.09). The median score for all medical articles (n=300) was 95.07 (95% CI 94.86-95.27).

#### Clout

##### Overview

**Figure 4:**
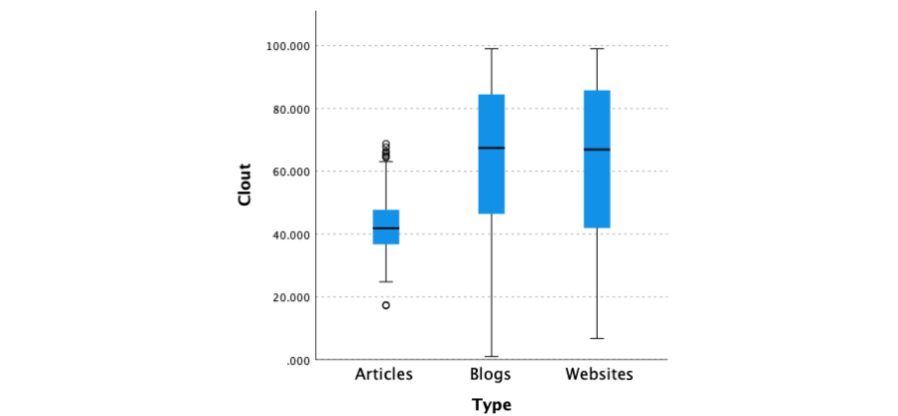
Linguistic Inquiry and Word Count clout scores by information type.

The median clout score for all 3 types of data (N=1139) was 53.68 (95% CI 51.04-55.73; Figure 4). There was a significant difference (*P*<.001) in the median clout scores across the 3 types of data, namely blogs, websites, and medical articles, with medical articles having significantly lower clout scores (*P*<.001, adjusted by the Bonferroni correction; median 41.86, 95% CI 40.31-43.06) than blogs (median 67.4, 95% CI 63.47-69.82) and websites (median 66.915, 95% CI 62.36-70.12). There was no significant difference in clout score between websites and blogs (*P*=.54, adjusted using the Bonferroni correction).

##### Websites

The median clout score for websites (n=612), including all subcategories, was 66.915 (95% CI 62.36-70.12). The distribution of the clout scores of websites was significantly different across target audiences, as evidenced by an independent sample Kruskal-Wallis test with a *P* value <.001. Websites targeted at caregivers (74/612, 12.1%) had significantly higher (*P*<.001, adjusted using the Bonferroni correction) clout scores (median 76.65, 95% CI 70.96-83.12) than those targeted at a general audience (median 61.66, 95% CI 51.0-69.12; 139/612, 22.7%) and those targeted at people with dementia (median 64.99, 95% CI 58.04-70.03; *P*<.001; 399/612, 65.2%). There was no significant difference in clout scores between information targeted at people with dementia and information targeted at a general audience (*P*=.99, adjusted using the Bonferroni correction).

##### Blogs

The distribution of the clout scores of blogs was not significantly different across the 3 target audiences, as confirmed by an independent sample Kruskal-Wallis test (*P*=.98). The median score for all blogs (n=227) was 67.4 (95% CI 63.47-69.82).

##### Articles

The median clout score for articles (n=300) was 41.86 (95% CI 40.31-43.06). The distribution of the clout scores of medical articles was significantly different between the 2 article types, as evidenced by an independent sample Mann-Whitney *U* test with a *P* value <.001. Articles with more biological and neuroscience content (72/300, 24%) had lower clout scores (median 39.25, 95% CI 36.97-41.180) than other clinical articles (median 42.27, 95% CI 41.15-44.22; 228/300, 76%).

#### Authenticity

##### Overview

**Figure 5:**
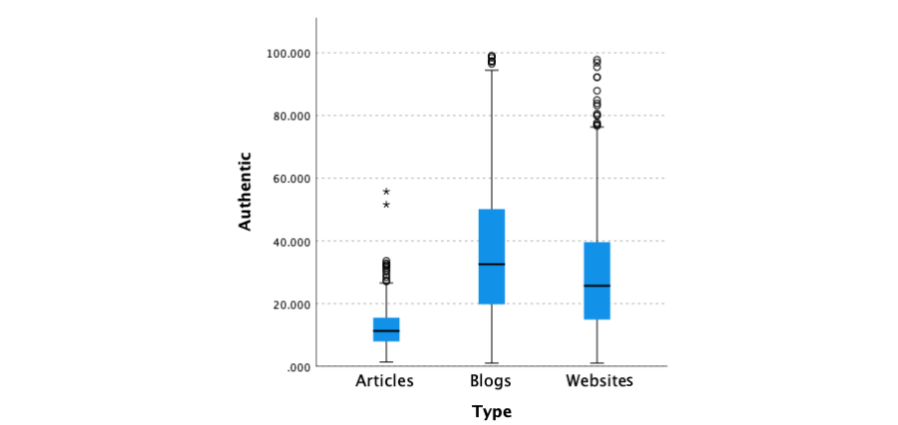
Linguistic Inquiry and Word Count Authentic scores by information type.

The median authenticity score for all 3 categories of data was 20.67 (95% CI 19.48-22.19; Figure 5). There was a significant difference (*P*<.001) in the median authenticity scores across the 3 types of data, namely blogs, websites, and medical articles, with medical articles having significantly lower (*P*<.001, adjusted by the Bonferroni correction) authenticity scores (median 11.275, 95% CI 10.69-12.57) than blogs (median 32.54, 95% CI 28.9-36.41) and websites (median 25.6, 95% CI 23.92-26.985). In addition, websites had significantly lower authenticity scores than blogs (*P*<.001, adjusted using the Bonferroni correction).

##### Websites

The median authenticity score for all websites (n=612; across all target audiences) was 25.70 (95% CI 23.92-26.98). A Kruskal-Wallis test indicated a significant difference (*P*=.006) in the median authenticity scores among the websites targeted at the 3 different audiences. Websites targeted at caregivers (74/612, 12.1%) had significantly lower authenticity scores (median 19.97, 95% CI 16.85-22.74) than those targeted at people with dementia (median 26.48, 95% CI 24.41-29.44; 399/612, 65.2%). There was no significant difference in authenticity scores between information targeted at people with dementia and information targeted at a general audience (median 25.92, 95% CI 22.93-30.98; *P*>.99, adjusted using the Bonferroni correction) or between information targeted at caregivers and information targeted at a general audience (*P*=.06, adjusted using the Bonferroni correction).

##### Blogs

The distribution of the authenticity scores of blogs was not significantly different across the 3 target audiences, as confirmed by an independent sample Kruskal-Wallis test (*P*=.36). The median score for all blogs (n=227) was 32.54 (95% CI 28.9-36.41).

##### Articles

The distribution of the authenticity scores of medical articles was not significantly different between the 2 article types, as confirmed by an independent sample Mann-Whitney *U* test (*P*=.44). The median score for all articles (n=300) was 11.275 (95% CI 10.69-12.57).

#### Emotional Tone

##### Overview

**Figure 6:**
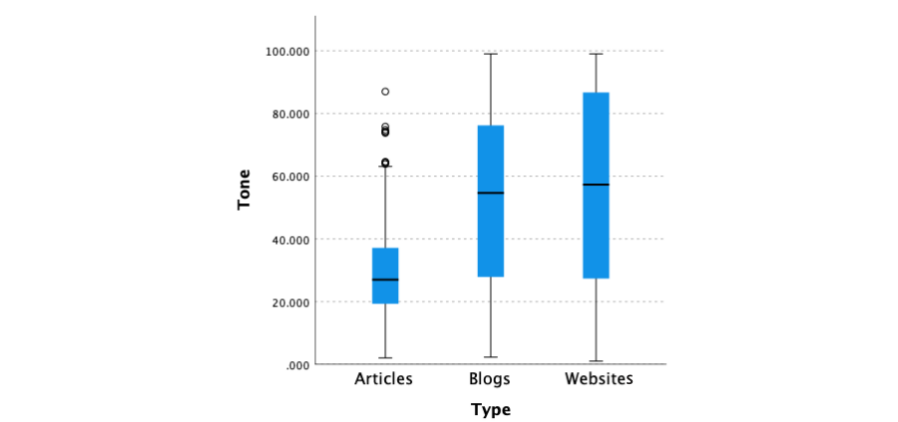
Linguistic Inquiry and Word Count emotional tone scores by information type.

The median analytical thinking score across all 3 information types (websites, blogs, and articles) was 41.42 (95% CI 39.22-45.82; Figure 6). There was a significant difference (*P*<.001) in the median emotional tone scores across the 3 types of information, with medical articles having significantly lower (*P*<.001, adjusted by the Bonferroni correction) tone scores (median 26.995, 95% CI 24.9-28.92) than blogs (median 54.64, 95% CI 48.58-59.02) and websites (median 57.305, 95% CI 52.18-61.44). There was no significant difference in tone scores between websites and blogs (*P*>.99, adjusted using the Bonferroni correction).

##### Websites

A Kruskal-Wallis test indicated a significant difference (*P*=.02) in the median tone scores among websites targeted at the 3 different audiences. Websites targeted at people with dementia (399/612, 65.2%) had significantly lower tone scores (median 55.66, 95% CI 47.86-60.85) than those targeted at caregivers (median 74.69, 95% CI 61.15-85.41; 74/612, 12.1%). There was no significant difference in emotional tone scores between information targeted at people with dementia and information targeted at a general audience (*P*>.99, adjusted using the Bonferroni correction) or between information targeted at caregivers and information targeted at a general audience (*P*=.06, adjusted using the Bonferroni correction).

##### Blogs

The distribution of the tone scores of blogs was not significantly different across the 3 target audiences, as confirmed by an independent sample Kruskal-Wallis test (*P*=.96). The median score for all blogs (n=227) was 54.64 (95% CI 48.58-59.02).

##### Articles

The median tone score for all articles (n=300) was 26.99 (95% CI 24.90-28.92). The distribution of the tone scores of medical articles was significantly different across the 2 article types, as evidenced by an independent sample A Mann-Whitney *U* test with a *P* value of .006. Articles with more biological and neuroscience content (72/300, 24%) had significantly lower tone scores (median 23.95, 95% CI 21.16-27.13) than other clinical articles (median 28.78, 95% CI 25.62-31.65; 228/300, 76%).

## Discussion

### Principal Findings

In this study, we conducted a multitrait, multimethod textual analysis of digital dementia information to investigate the readability and linguistic characteristics of different information types, namely websites, blogs, and medical articles. Using thematic analysis, we found that most of the blogs (156/227, 68.7%) and websites (399/612, 65.2%) were targeted at people with dementia. In addition, we found that website information targeted at a general audience had significantly lower readability scores than information targeted at people with dementia or caregivers. Further, websites targeted at people with dementia had longer word counts and lower emotional tone scores than websites targeted at caregivers or a general audience. In addition, the information on websites targeted at caregivers had significantly higher clout scores and lower authenticity scores than that on websites targeted at people with dementia or a general audience. Finally, we found that medical articles with more biological and neuroscience terminology were less readable and had lower clout and emotional tone scores than those with less medical terminology.

### Implications

#### Information Target Audience

The data showed that most of the content directed at people with dementia was found on blogs, accounting for 159 (70%) out of 227 blogs. This finding is not surprising, as most blogs were written by individuals with dementia about their individual experiences to assist others in similar circumstances. Similarly, the largest proportion of websites was also directed at people with dementia, representing 399 (65.2%) out of 612 websites. Despite the abundance of content available on the web, past studies have reported that people with dementia may still struggle to find and access relevant digital information [8,13]. This finding provides further support to past studies that report that the challenge with digital dementia information may largely be due to the accessibility of information rather than the lack of available content.

#### Readability, Word Count, and Analytical Thinking: Indicators of Access

Past contextual inquiry studies have demonstrated that people with dementia often resort to using information shared by others living with the condition rather than other information types (websites) owing to a lack of accessibility of the latter [8,32]. Our findings provide evidence supporting these past studies, revealing that none of the 3 types of information sources analyzed (websites, blogs, and articles) met the recommended minimum standards for readability, which are a Flesch Reading Ease score of 50 and a Flesch-Kincaid Grade Level of seventh to eighth grade [65,100]. When considering that people with <12 years of education [42,123] and those with lower eHealth literacy [48] are at a higher risk of developing dementia, our findings indicate that individuals with an education level <11th grade would have difficulty reading even blogs; therefore, a large portion of the people who need this information may not be able to read it.

Some contributing factors to these low readability scores for medical articles may include word count and analytical thinking scores, as medical articles had significantly higher median word count and analytical thinking scores. Further, articles that used more medical terminology had significantly lower readability scores than those that did not. These findings were expected, given that medical articles are often written for a target audience of other academic researchers. However, these findings also present a disconnect between the desire and need of people with dementia and informal caregivers to be informed of the latest breakthroughs in dementia research by searching for academic research on the web [8,13,20,21,39] and the readability of this information.

In addition, our findings indicated that web pages had fewer word counts but higher analytical thinking scores than blogs. Despite having lower word counts, websites did not meet the minimum readability scores, whereas the readability of blogs was just under the mark. Further, web pages targeted at people with dementia had a significantly higher word count (536) than those targeted at a general audience (353) and those targeted at caregivers (343). These findings are problematic, given that past work [32,124] has shown that people with dementia have difficulty reading and comprehending lengthy or complex information.

Considering that the findings of the study indicate a deficiency in the readability of digital dementia information, we join past studies in urging content creators to improve the readability of their content [8,32], developing resources that are responsive to the low eHealth literacy levels of this population [46]. As a starting point to accomplishing this goal, we suggest following governmental plain language guidelines [125], the World Wide Web Consortium’s Cognitive and Learning Disabilities Accessibility Task Force’s recommendations for “Making Content Usable for People with Cognitive and Learning Disabilities” [126], and the Dementia Engagement and Empowerment Projects’ “Guidelines on language about dementia” [126,127]. On the basis of the findings of this study, we specifically suggest that content creators follow these recommendations [27,64,126].

#### Clout and Authenticity: Indicators of Persuasion and Trust

The clout analysis showed that blogs had the highest clout scores (median 67.40), followed closely by websites (median 66.91), whereas medical articles had the lowest clout scores (median 41.86). A high median score in clout measurement is an indication that the language used in the text conveys a sense of social status or power, which may be perceived by the audience as authoritative or influential. Within the web pages analyzed, we found that information targeted at caregivers had the highest clout score (median 76.65), suggesting that the language used toward caregivers on websites conveys a sense of social status or power. Therefore, caregivers accessing digital dementia information with higher clout scores may perceive their role and responsibilities in a more authoritative manner, which could have implications for their caregiving practices. Considering the relational dynamics that change between spouses and parents or children’s relationships after someone is diagnosed with dementia [19,128-132], this information can either be perceived as empowering caregivers to provide the best care they can or lead to a more authoritative approach that can be demeaning and borderline abusive to people with dementia [40].

The authenticity scores of the data collected in this study were relatively low; the authenticity scores of medical articles (median 11.275) were significantly lower than those of blogs (median 32.54) and websites (median 25.7). Given that all authenticity scores were relatively low, this suggests that the information, particularly medical articles and websites, may not be perceived as “honest,” “real,” or “true.” Instead, these low authenticity scores indicate the information implies deception or insincerity, with words such as “fake,” “pretend,” and “lie” contributing to a low score.

These findings have implications for the perceived trustworthiness of information provided by these platforms. For example, readers may perceive blog content as more confident, assertive, and authentic, which could influence their decision-making process. Considering the inaccuracy of digital dementia information authored by nonmedical professionals [28,29,44], this could lead to misplaced trust in strategies and therapeutic techniques suggested in blogs that may not be validated. Further, with past research associating lower eHealth literacy with lower caregiving self-efficacy [45] and, consequently, lower health outcomes for the associated people with dementia [47], these findings highlight the necessity of future research investigating the design of systems to indicate the reliability and accuracy of digital dementia information.

#### Emotional Tone: Indicator of Mental Health Effects

The overall median emotional tone score for all platforms was 41.42, an average-to-low emotional score. Our findings indicate that medical articles had significantly lower emotional tone scores than websites and blogs, indicating that medical articles include significantly fewer emotions (whether positive or negative in their text). This may be attributed to the assumption that the target audience of medical articles would be academic researchers rather than people with dementia or caregivers, although past work has shown that people with dementia and caregivers also interact with this information [8].

Nearly all websites (578/612, 94.4%) and all blogs (227/227, 100%) contained words categorized as emotionally negative words. By contrast, only 55.4% (339/612) of websites and 85% (193/227) of blogs contained words categorized as emotionally positive. Further, when investigating the frequency of content subcategories, we found that 99.1% (225/227) of blogs and 98% (600/612) of websites used words related to illness, whereas only 45.8% (104/227) of blogs and 41% (251/612) of websites used words related to wellness. In addition, the percentages of content related to “anxiety,” “anger,” “sadness,” and “death” on blogs were nearly double those on websites (eg, 60% of blogs contained words related to anxiety, whereas 36% of websites contained words related to anxiety).

These findings provide evidence supporting past studies that report overwhelmingly negative dementia information [8,32,33] and the need for more emotionally accessible digital dementia information [32]. The overwhelmingly negative digital dementia information may be a contributing factor to the mental health [61] crisis many people with dementia face after receiving a diagnosis; people diagnosed with dementia who were aged <65 years were 6 times more likely than people of the same age without dementia [133] and 3 times more likely than people diagnosed with dementia who were aged >75 years [117] to die by suicide within 3 months of their diagnosis. To address this concern, future research in collaboration with dementia advocacy groups (eg, DEEP [134]) is needed to investigate ways in which information resources could be designed to avoid the “doom and gloom” narrative surrounding dementia while still providing accurate and reliable information.

### Limitations and Future Work

There were limitations to the LIWC analysis method, as LIWC has 80 categories, of which we focused on 5 main categories and the subcategories related to emotions and tone. We excluded categories such as pronouns, family, friends, and wealth. Another limitation of LIWC is its scoring mechanism; given that it relies solely on linguistic features, it may not always accurately capture the true linguistic characteristics of an author’s writing (eg, intended authenticity). As this research specifically concentrated on LIWC analysis, vocabulary density was not incorporated. However, this aspect could be considered in future investigations. Although we provide frequencies of subcategories, they alone do not provide a complete picture of the tone or sentiment conveyed in the text. For instance, if a blog post or an article frequently uses negative words such as “death” and “died” in reference to individuals with dementia or their caregivers, it may create a somber or depressing tone for the reader. By contrast, if the same words are used in a positive or an empowering context, such as when discussing the resilience of caregivers in the face of loss or the impact of a person’s legacy after they have passed, the emotional tone may be more uplifting.

The scope of the thematic analysis is also limited in that we did not analyze the dates of publications of different informational sources to ascertain the temporal trends in the number of dedicated dementia websites or blogs, an area that remains open for exploration. Further, the thematic analysis included in this study did not attempt to assess the quality or accuracy of the information, although these factors are also major determinants of the usefulness of web-based dementia information [32]. Future work should consider taking a collaborative content analysis approach, involving individuals living with dementia, active informal caregivers, and medical professionals as collaborators. This would afford the opportunity to not only assess the quality of the information but also yield a more nuanced understanding of the sentiment and thematic elements conveyed in the content, enabling researchers and analysts to draw more informed conclusions.

This study is also limited in that we focused on purely text-based content. Areas open for future investigation include the accuracy, quality, and accessibility of existing multimedia information formats, such as videos and infographics, as well as ways to design future tangible or interactive displays to deliver dementia-related information that is accurate, cognitively and emotionally accessible, and captivating for the target audience. Further, building on previous work [135], future work in this area could investigate how different types of multimedia dementia content influence people’s perceptions, attitudes, and stereotypes surrounding dementia.

### Conclusions

To explore the linguistic, psychological, and emotional traits of digital information on dementia, we conducted a multitrait, multimethod textual analysis of 3 distinct types of text-based sources: 300 medical research articles on dementia published in the last 5 years; 35 dementia-focused advocacy and medical organization websites; and 50 dementia blog sites. We evaluated the readability of a given text using the Flesch-Kincaid Grade Level and Flesch Reading Ease measurements. In addition, we used the NLP tool LIWC to analyze word count, analytical thinking, clout, authenticity, emotional tone, and word frequencies.

Our findings indicate that there is an abundance of digital dementia information targeted at people with dementia, with most blogs (156/227, 68.7%) and websites (399/612, 65.2%) targeted at people with dementia. However, the readability scores of all 3 platforms (advocacy websites, blogs, and medical articles) did not meet the minimum readability threshold of a seventh to eighth grade reading level, with blogs being the most readable at an 11th grade level, indicating a difficulty in comprehending the information presented. This is problematic when considering that people with <12 years of education and lower eHealth literacy levels are at a higher risk of developing dementia. We suggest that creators of digital dementia content increase the readability of their content to make it more accessible to a general audience.

Medical articles had higher word counts and analytical thinking scores but lower clout, authenticity, and emotional tone scores than websites and blogs. Further, we found that blog content was written in a more confident, assertive, and authentic manner than websites or articles, which could influence readers’ decision-making and lead to misplaced trust in strategies and therapeutic techniques that are not validated. In addition, we found that websites targeted at caregivers had higher clout scores, meaning that readers may perceive the caregiving role and responsibilities in a more authoritative manner, which could have implications for their caregiving practices.

Finally, the sentiment analysis indicated that digital dementia information has a negative tone, which may be a contributing factor to the mental health crisis many people with dementia face after receiving a diagnosis. Therefore, we urge content creators to focus efforts on providing information in a way that does not perpetuate the overly negative narrative surrounding dementia, particularly if the content is created for readers with dementia.

## Abbreviations

LIWC: Linguistic Inquiry and Word Count
NLP: natural language processing

## Data Availability

The data sets that were both generated and analyzed as part of this study were curated and are accessible. Anyone interested in obtaining these data sets can approach the corresponding author and submit a request. The corresponding author serves as the point of contact for such inquiries and will facilitate the process of making these data sets available to those submitting a request. This approach ensures that valuable research resources are shared in a manner that promotes collaboration, transparency, and the advancement of scientific knowledge.
